# Predictors for long-term outcome of pulmonary valve perforation and balloon valvuloplasty in neonates with critical pulmonary valve stenosis or pulmonary valve atresia with intact ventricular septum

**DOI:** 10.3389/fcvm.2025.1527832

**Published:** 2025-03-12

**Authors:** Tariq Abu-Tair, Ines Willershausen, Melanie Friedmann, Kai Rubarth, Annika Weigelt, Claudia Martin, Sven Dittrich, Christoph Kampmann

**Affiliations:** ^1^ Department of Congenital Heart Diseases, Centre for Diseases in Childhood and Adolescence, University Medicine Mainz, Mainz, Germany; ^2^ Department of Pediatric Cardiology, Friedrich-Alexander-University Erlangen-Nürnberg, Erlangen, Germany; ^3^ Department of Orthodontics and Orofacial Orthopedics, Friedrich Alexander-University Erlangen-Nürnberg, Erlangen, Germany

**Keywords:** outcome, balloon valvuloplasty, pulmonary valve, pulmonary atresia, pulmonary stenosis

## Abstract

**Introduction:**

Percutaneous balloon valvuloplasty is the treatment of choice for critical pulmonary valve stenosis (CPS) and pulmonary valve atresia with intact ventricular septum (PA/IVS) if the ventricle has a suitable size. This study aimed to evaluate the long-term outcomes and predictors for surgical intervention and pulmonary valve replacement in patients with CPS or with PA/IVS after PBV, considering different morphological and hemodynamic parameters.

**Methods:**

Neonates with PA/IVS or CPS who were admitted to the University Medicine Mainz and University Hospital Erlangen between November 1994 and March 2013 and underwent successful PBV as an initial procedure, with a follow-up of at least 5 years (median 13.1 years), were included. The *Z*-scores of pulmonary valve diameter, balloon/annulus ratio, number of cusps, and persisting stenosis were analyzed. The endpoint was the need for surgical procedures or valve replacement.

**Results:**

A total of 62 neonates (median age at intervention 5 days) were included. Among them, 15 patients (24.2%) reached the endpoint. The mean time of freedom from surgery differed according to the number of cusps (*P* < 0.001), pulmonary valve diameter *Z*-scores (*P* = 0.04), and degree of persisting stenosis (*P* = 0.008), but did not differ according to the balloon/annulus ratio (≤1.2 vs. >1.2).

**Conclusion:**

Pulmonary valve perforation and PBV achieved favorable long-term outcomes in neonates with PA/IVS and CPS. A small pulmonary valve diameter, reduced number of cusps, and persisting gradient of >40 mmHg increased the risk for reduced time of freedom from surgical intervention and/or pulmonary valve replacement.

## 1 Introduction

Congenital heart defects are the most frequent organ malformations in humans, occurring in 3–13 per 1,000 live births (1). Critical pulmonary valve stenosis (CPS) and pulmonary valve atresia with intact ventricular septum (PA/IVS), which account for approximately 2% of all congenital heart defects, are a group of rare and heterogeneous abnormalities that differ in the morphology of the pulmonary valve (PV), tricuspid valve (TV), and right ventricle (RV). The pathology of CPS and PA/IVS is life-threatening, and if left untreated, affected patients will have a severely reduced life expectancy due to right-to-left shunting at the level of the foramen ovale and duct-dependent perfusion of the pulmonary artery. This leads to central cyanosis and/or systemic hypoxemia, necessitating immediate treatment (2). Historically, pulmonary perfusion in patients with CPS and PA/IVS was first augmented by the implantation of a Blalock–Taussig shunt (3). The introduction of cardiopulmonary bypass enabled the surgical opening of the right ventricular (RV) outflow tract using the Brock procedure, valvulotomy, or a transannular patch (4). However, all of these surgical modalities are associated with high morbidity and mortality (5). In 1982, Kan et al. (6) introduced the percutaneous balloon valvuloplasty (BV) technique as an interventional strategy for congenital PV stenosis. This advanced technique was first performed in infants by Tynan et al. (7) in 1984. Since then, transcatheter PV perforation followed by balloon valvuloplasty (BV) has become the gold standard for the early treatment of CPS and PA/IVS in neonates (8–10).

PBV is a safe and effective procedure for establishing antegrade pulmonary perfusion. The development of new catheterization techniques (e.g., manual, radiofrequency, or laser-assisted valve perforation), new materials, and skilled post-interventional care have resulted in improved prognoses. Immediate and major complications include cardiac or primary pulmonary artery perforation (11). Long-term adverse effects include the need for repeated percutaneous interventions for restenosis or PV replacement in patients with severe pulmonary regurgitation, and the development of progressive PV regurgitation or persistent RV outflow tract obstruction is considered the primary indication for repeat interventions. PV regurgitation is a severe condition that affects a limited number of children undergoing BV. Large clinical studies have focused on assessing the post-interventional development of progressive pulmonary valve regurgitation. Persisting PV stenosis is rarer, and data on patient follow-ups are scarce (12, 13). This might be because of the heterogeneity of the patient group, the rarity of these conditions, and the need for a long follow-up period as PV regurgitation progresses slowly. Therefore, the present study assessed the preconditioning/risk factors associated with the development of severe PV regurgitation leading to PV replacement after BV or severe persistent stenosis leading to surgical reintervention in patients with CPS and PA/IVS.

## 2 Materials and methods

### 2.1 Ethics

The ethics boards of the University Medicine Mainz, Germany, and the University Hospital Erlangen, Germany, waived the need for approval due to the retrospective nature of the analyses and the analysis of previously collected data from hospital databases.

### 2.2 Study design and patients

This retrospective study evaluated neonates who presented with CPS or PA/IVS at the Department of Congenital Heart Disease, Center for Diseases in Childhood and Adolescence, University Medicine, Mainz, Germany, and the Department of Pediatric Cardiology at the University Hospital in Erlangen, Germany, between November 1994 and March 2013 undergoing balloon valvuloplasty of the pulmonary valve. Data collection began in 2018, with an observation period of at least 5 years to evaluate long-term outcomes. Patients with additional congenital cardiac malformations, such as ventricular septal defects, which would require further surgical interventions, were excluded. Patients converted to single ventricle palliation or 1.5-ventricle palliation were excluded due to early surgical procedures involving shunt or Glenn anastomosis without the focus on pulmonary valve surgery. CPS was defined as suprasystemic RV pressure and/or right-to-left shunting across the patent foramen ovale/atrial septal defect and/or duct-dependent pulmonary blood flow, as verified by pre-interventional echocardiography. To be included, patients needed to fulfill at least two of these criteria.

### 2.3 Endpoint and data collection

The endpoint was defined as the need for surgical intervention, e.g., PV replacement or procedures such as PV valvulotomy, transannular patch, monocusp right ventricular outflow tract (RVOT) repair, or PV commissurotomy. Baseline biometric data on age, sex, weight, height, and body surface area were recorded. Additionally, angiographic and procedural data related to cardiac morphology and function were collected, pre-intervention, post-intervention, and at follow-up. Angiographic parameters included the morphology of the right ventricle in the lateral projection, which was defined according to the TV annulus, diastolic RVOT, PV annulus, and main pulmonary artery diameters.

### 2.4 Measurements

The *Z*-scores of the TVs and PVs and the TV and PV diameters were calculated according to Zilberman et al. (14), and those of the main pulmonary artery and main pulmonary artery diameter were calculated according to Pettersen et al. (15). The morphology of the PV was assessed during cardiac catheterization using RV angiography in the frontal projection and antegrade pulmonary artery angiography or ductus angiography. The PV was subsequently subdivided represented by the number of leaflets and raphes in tricuspid, bicuspid, or monocusp valves, which subsumed the pinhole morphology. Leaflets were considered even when they were fused and divided solely by the raphes. For example, if one leaflet was divided by a raphe, it was considered as two leaflets, and if an atretic valve had three raphes, it was classified as a tricuspid. The balloon–annulus ratio (BAR) during the intervention was recorded. Systolic and end-diastolic RV pressure before, during, and after catheterization was measured.

### 2.5 Procedural protocol

The procedure was performed under conscious sedation with esketamine, midazolam, and propofol, or general anesthesia with mechanical ventilation during the procedure, for patients with PV atresia. Vascular access was achieved by puncturing the femoral artery and vein or via the umbilical vein. Arterial blood pressure, heart rate, respiratory frequency, and oxygen saturation were continuously monitored. Prostaglandin E1 was administered in patients with duct-dependent pulmonary perfusion. Biplane angiography of the right ventricle in the frontal and lateral projections was performed to evaluate the PV, anatomy of the right ventricle, and the hemodynamic effect of pulmonary stenosis/atresia. In the frontal projection, the morphology of the PV and the number of leaflets were obtained. In the lateral projection, the residual outflow of the right ventricle in the pulmonary artery was differentiated.

Subsequently, the stenotic valve was passed through, while atretic valves were either mechanically or electrically perforated. Balloon valvuloplasty was performed with semi-compliant balloons such as the Tyshak II balloon (pfm medical gmbH, Cologne, Germany) or the VACS II balloon (Osypka GmbH, Rheinfelden, Germany). Finally, hemodynamic evaluation and angiography of the pulmonary artery were performed. Prostaglandin E1 infusion was discontinued or tapered according to oxygen saturation. The outcomes of balloon valvuloplasty were evaluated using echocardiographic examination at the outpatient follow-up at 1, 3, and 6 months post-intervention and then annually. In addition, tricuspid and PV regurgitation and residual PV stenosis were identified and analyzed. In patients with severe PV regurgitation, magnetic resonance imaging was used to evaluate the need for PV replacement based on echocardiographic examination according to the German Society of Pediatric Cardiology guidelines and for residual pulmonary stenosis, which may require surgical intervention, according to the guidelines of the German Society of Pediatric Cardiology in pulmonary valve stenosis.

### 2.6 Statistical analysis

Continuous demographic and procedural data are presented as the median and standard deviation. Comparisons between two and three groups were performed using the Mann–Whitney *U*-test and the Kruskal–Wallis test, respectively. Kaplan–Meier curves were generated and compared using Mantel–Cox analysis. All statistical analyses were performed using IBM SPSS Statistics Version 29.0.0.0 for Windows software (Chicago, IL, USA). A *p*-value of 0.05 was considered statistically signiﬁcant.

## 3 Results

### 3.1 Patient characteristics

The sample comprised 62 patients (34 females). The median patient age at intervention was 5 days with a range from 1 to 60 days, the median weight was 3.3 ± 0.6 kg, the median height was 51 ± 3.7 cm, and the body surface area was 0.21 ± 0.03 m^2^. Patients were followed up to 24.6 years (median, 13.1 years; range, 5–24.6 years) after the initial intervention. No patient died during follow-up. Hemodynamic and morphological data of all patients are presented in Tables 1 and 2.

**Table 1:** Hemodynamic data (*n* = 62).

Measure	Statistics	Value
Systolic right ventricular pressure, pre-intervention (mmHg)	x̅ ± SD Median Range	97 ± 23 95 (52–169)
Diastolic right ventricular pressure, pre-intervention (mmHg)	x̅ ± SD Median Range	6.8 ± 5 6 (0–20)
Systolic right ventricular pressure, post-intervention (mmHg)	x̅ ± SD Median Range	55 ± 19 51 (27–128)
Diastolic right ventricular pressure, post-intervention (mmHg)	x̅ ± SD Median Range	5 ± 3.8 5 (0–15)
Systolic pulmonary arterial pressure, post-intervention (mmHg)	x̅ ± SD Median Range	35 ± 12 34 (16–76)
Diastolic pulmonary arterial pressure, post-intervention (mmHg)	x̅ ± SD Median Range	15 ± 9 14 (1–54)
Systolic left ventricular pressure, pre-intervention (mmHg)	x̅ ± SD Median Range	71 ± 13 69 (46–110)

**Table 2:** Morphologic and procedural data (*N* = 62).

Measure	Statistics	Value
Main pulmonary artery diameter (mm)	x̅ ± SD Median Range	8.7 ± 2.3 8.5 (4.2–15.2)
Main pulmonary artery *Z*-score	x̅ ± SD Median Range	0.3 ± 1.8 0.1 (−2.9 to 4.65)
Pulmonary valve diameter (mm)	x̅ ± SD Median Range	6.8 ± 1.4 6.8 (4–11.3)
Pulmonary valve diameter *Z*-score	x̅ ± SD Median Range	−1.6 ± 1.6 −1.5 (−4.98 to 3.44)
Right ventricular outflow tract diameter (mm)	x̅ ± SD Median Range	7.1 ± 2.4 6.9 (3.6–15.8)
Tricuspid valve diameter (mm)	x̅ ± SD Median Range	10.9 ± 2.6 11.1 (5–18.2)
Tricuspid valve *Z*-score	x̅ ± SD Median Range	−0.5 ± 1.6 −0.6 (−4.55 to 4.08)
Balloon–annulus ratio	x̅ ± SD Median Range	1.1 ± 0.2 1.1 (0.69–1.73)

In 18/62 patients (29%), a second cardiac catheterization with additional balloon valvuloplasty of the pulmonary valve has been performed of whom 8/18 (44%) received surgical intervention. In 5/62 patients (8%) who have undergone three cardiac catheterizations, 3/5 (60%) received surgical intervention. In 4/62 patients, ductal stenting has been performed. In three of these patients, the ductus closed due to neointimal hyperproliferation, and in one patient, it closed during early valve replacement.

A total of 15 (24.2%) patients reached the endpoint during follow-up and required Contegra (Medtronic GmbH, Meerbusch, Germany) PV replacement (*n* = 6), monocusp RVOT repair (*n* = 2), transannular RVOT patch repair (*n* = 5), SAPIEN (Edwards Lifesciences GmbH, Garching, Germany) transcatheter valve replacement (*n* = 1), and PV commissurotomy (*n* = 1).

### 3.2 Outcomes according to the number of valve leaflets

**Figure 1:**
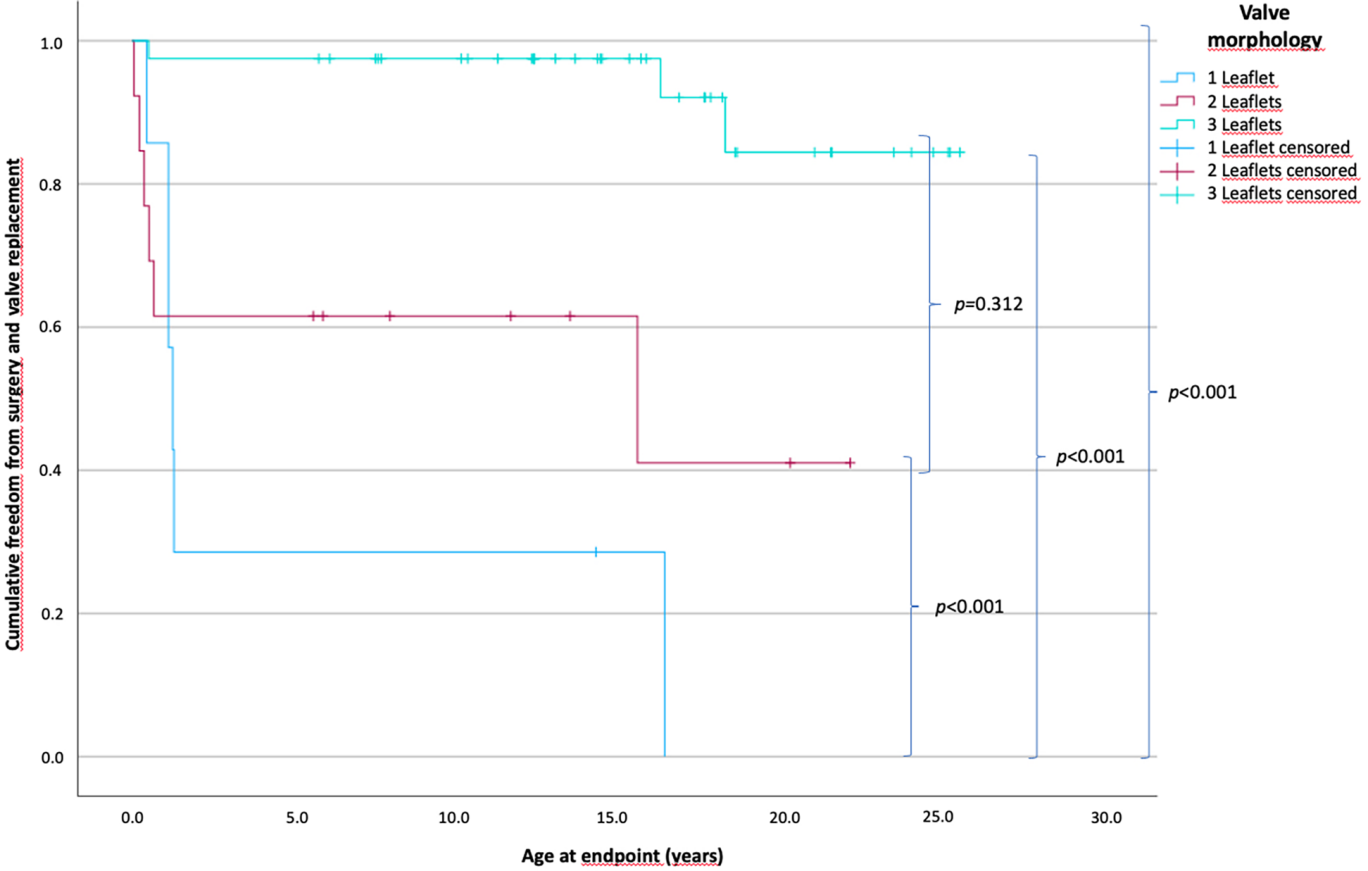
Patients’ cumulative freedom from surgery according to the number of pulmonary valve leaflets. *p*-values of differences between the groups are in curly brackets; there is a statistically significant difference between all groups of *p* < 0.001. The mean freedom between groups is 23.9 years for tricuspid valves, 12.5 years for bicuspid valves, and 5.5 years for monocuspid valves (one leaflet, *n* = 7; two leaflets, *n* = 14; three leaflets, *n* = 41).

The patients were divided into three groups according to the number of valve leaflets. The monocuspid, bicuspid, and tricuspid valve groups comprised 7, 14, and 41 patients, respectively. The estimated time of freedom from surgery is shown in Figure 1. The mean time of freedom from surgery differed significantly between groups (*p* < 0.001) and was 23.9 years for tricuspid valves, 12.5 years for bicuspid valves, and 5.5 years for monocuspid valves.

In the monocuspid valve group, 6 of 7 patients (85.7%) (0.5–16.5 years); in the bicuspid valve group, 6 of 14 patients (42.9%) (0.1–15.6 years); and in the tricuspid valve group 3 of 41 patients (7.3%) (0.1–18.3 years) reached the endpoint and required surgery or transcatheter valve replacement.

### 3.3 Outcomes according to the *Z*-scores

**Figure 2:**
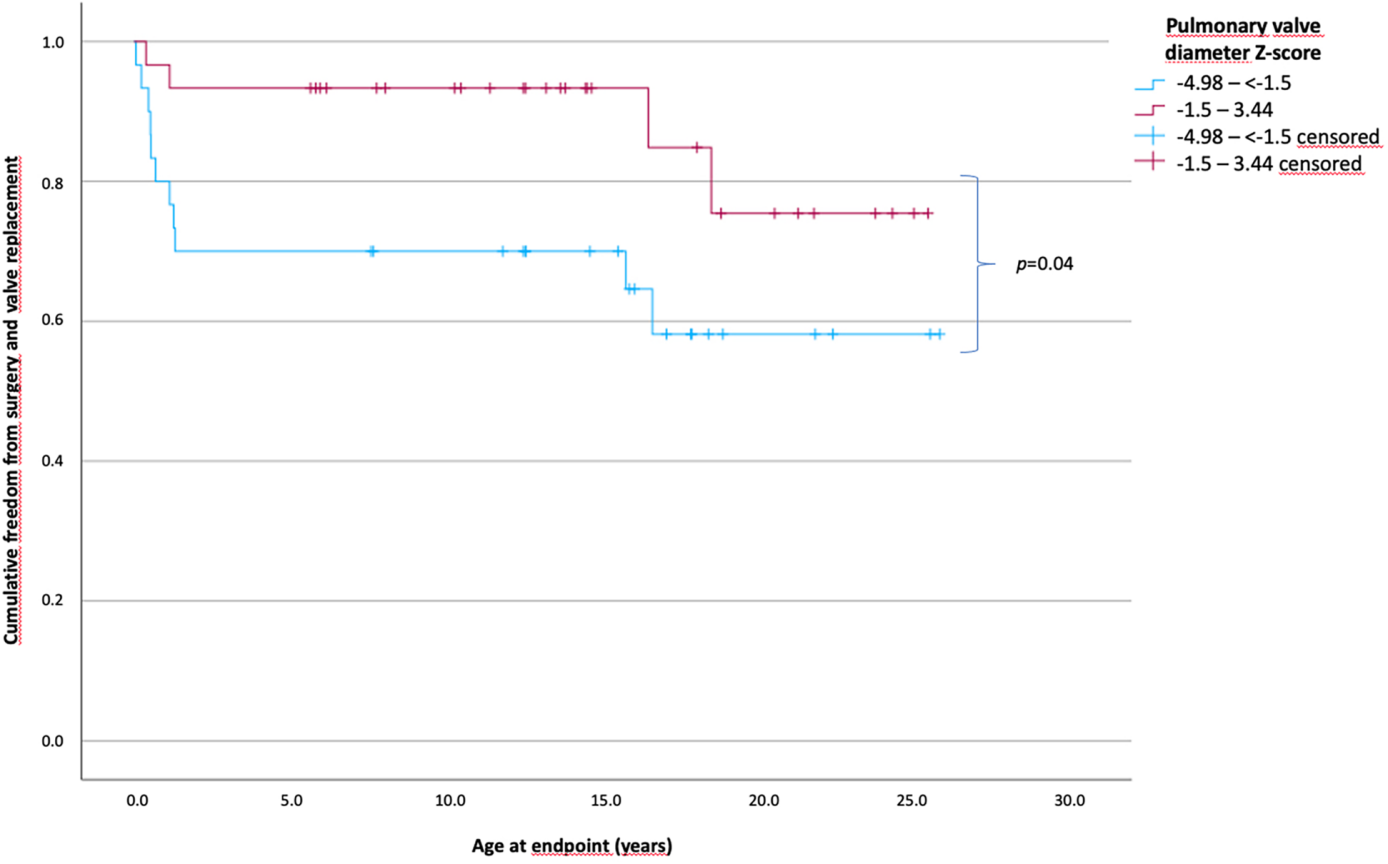
Patients’ cumulative freedom from surgery of patients according to the *Z*-scores of the pulmonary valve diameter. Patients divided into two groups according to the *Z*-scores of the pulmonary valve diameter less than −1.5 and ≥1.5. A significant difference was observed in long-term survival without the need of surgical intervention or valve replacement (*p* = 0.04). The mean freedom from surgery was 17 years in patients with *Z*-scores below −1.5 and 22.2 years in the patients with *Z*-scores of −1.5 and higher (*Z*-scores from −4.98 to less than −1.5, *n* = 31; *Z*-scores from −1.5 to 3.44, *n* = 31).

Overall, 31 patients had pulmonary valve diameter *Z*-scores between −4.98 and less than −1.5 and 31 patients between −1.5 and 3.44. The time of freedom from surgery according to the *Z*-scores of pulmonary valve diameter is shown in Figure 2. The mean time of freedom from surgery differed significantly between groups (*p* = 0.04) and was 17 years in patients with *Z*-scores below −1.5 and 22.2 years in patients with *Z*-scores of −1.5 and higher. In total, 11 patients (35.5%) [0.1–16.56 years] with *Z*-scores below −1.5 reached the endpoint, compared to 4 patients (12.9%) (0.2–18.3 years) with *Z*-scores of −1.5 and higher (*p* < 0.001). Morphological parameters as the RVOT diameter (mean, 6.1 vs. 8 mm; *p* = 0.008) and the *Z*-score of TV diameter (mean, −1.0 vs. 0.02; *p* = 0.024) were significantly lower in the group with a *Z*-score below −1.5. Furthermore, for procedural parameters, the BAR was significantly higher in those patients with *Z*-scores below −1.5 (mean, 1.2 vs. 1.07; *p* = 0.024).

### 3.4 Outcomes according to the ballon–annulus ratio

A balloon–pulmonary valve diameter ratio of ≤1.2 was used in 39 patients, and a ratio of >1.2 was used in 23 patients.

The PV diameter (mean, 7 vs. 6 mm; *p* = 0.009) and PV diameter *Z*-score (mean, −1.1 vs. −2.5; *p* = 0.002) were significantly smaller in the group with a BAR of >1.2.

**Figure 3:**
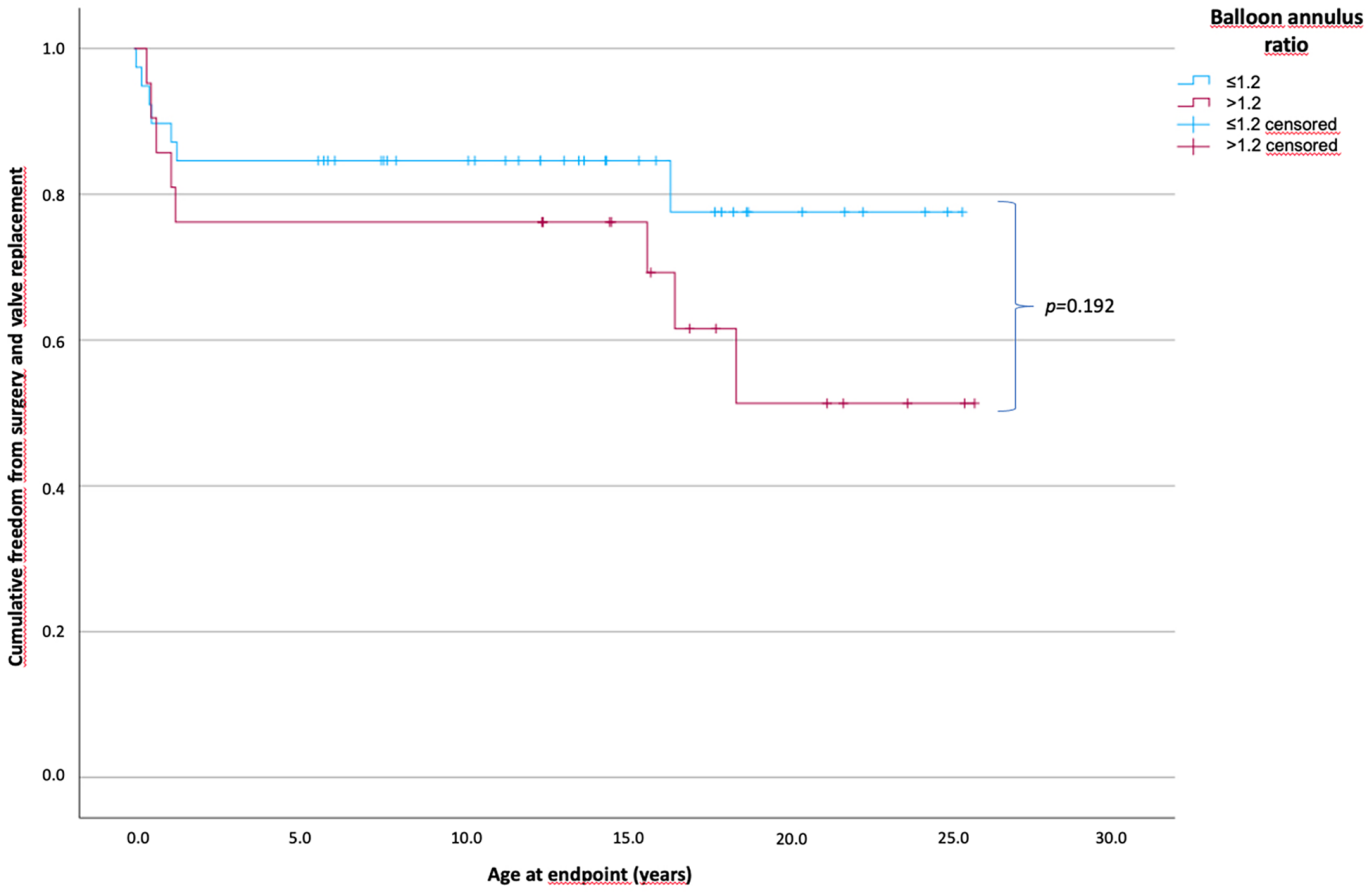
Cumulative freedom from surgery of the two patient groups according to the balloon–annulus ratio. Patients divided into two groups according to BAR of ≤1.2 and >1.2 showed no significant difference in long-term survival without the need for surgical intervention or valve replacement (*p* = 0.192). The mean freedom from surgery with a BAR of ≤1.2 was 20.8 years and 18 years for a BAR of >1.2. (BAR ≤1.2, *n* = 39; BAR >1.2, *n* = 23).

The time of freedom from surgery according to the BAR is shown in Figure 3. The mean time of freedom from surgery in the BAR ≤1.2 group was 20.8 years and 18 years in the BAR >1.2 group, and did not differ within the groups. However, in the BAR ≤1.2 group, 7 of 39 patients (17.9%) (0.1–1.1 years) reached the endpoint, while in the BAR >1.2 group, 8 of 23 patients (34.8%) (0.7–18.3 years) required surgery (*p* = 0.088).

### 3.5 Outcomes according to the persisting gradient across the RVOT after BV

With respect to the persisting gradient across the RVOT after BV, 36 patients had a residual gradient of ≤20 mmHg, 16 patients had a gradient from 21 to 40 mmHg, and 10 patients had a gradient of >40 mmHg. The hemodynamic, morphologic, and procedural parameters were not significantly different among the groups, except that the post-interventional RV systolic pressure significantly increased as the persisting gradient increased (mean, 44.5 mmHg for the ≤20 mmHg group vs. 63.3 mmHg for the 21–40 mmHg group vs. 83.4 mmHg for the >40 mmHg group; *p* < 0.001).

**Figure 4:**
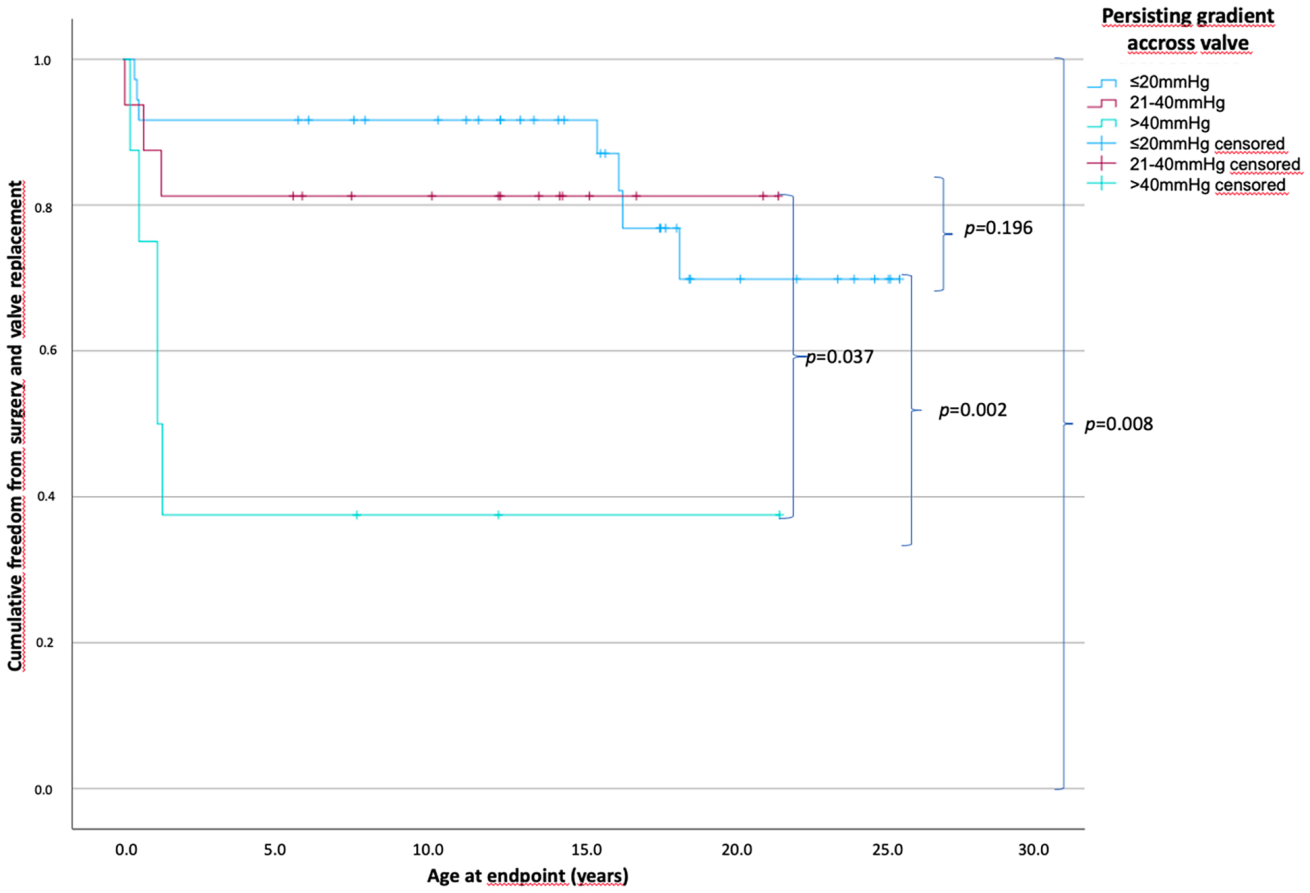
Patients’ cumulative freedom from surgery based on the persistent gradients across the pulmonary valve. *p*-values of differences between the groups in curly brackets. There is a statistically significant difference between all groups of *p* = 0.008 and no statistically significant difference between the group with a persistent gradient across the valve of ≤20 and 21–40 mmHg with a *p* = 0.196. The mean freedom from surgery differed significantly from 21.6, 17.7, and 8.6 years in patients with a gradient of ≤20, 21–40, and >40 mmHg, respectively (persisting gradient ≤20 mmHg, *n* = 36; 21–40 mmHg, *n* = 16; >40 mmHg, *n* = 10).

The time of freedom from surgery according to the persisting residual gradient across the RVOT is shown in Figure 4. The mean time of freedom from surgery differed significantly: 21.6 years in patients with a gradient of ≤20 mmHg, 17.7 years in patients with a gradient between 21 and 40 mmHg, and 8.6 years in patients with a gradient over 40 mmHg (overall *p* = 0.008), but not within the groups where the gradient was ≤20 and <40 mmHg (*p* = 0.196). Seven patients (18.9%) (0.1–18.3 years) in the ≤20 mmHg group reached the endpoint; three patients (18.8%) (0.1–1.5 years) in the 21–40 mmHg group, and five patients (55.6%) (0.2–1.1 years) in the cohort with a residual gradient above 40 mmHg.

## 4 Discussion

Transcatheter treatment of critical pulmonary valve stenosis and pulmonary atresia with intact ventricular septum and acceptable RV dimensions offers excellent long-term outcomes in favorable morphology and long survival times without the need for surgical intervention.

Nevertheless, predictors for early surgical intervention are dysplastic valves with ≤2 leaflets, a small pulmonary valve diameter, or a high persistent gradient across pulmonary valves of >40 mmHg.

Although balloon valvuloplasty of stenotic PV was initially described by Kan et al. (6) in 1982, advanced techniques were developed later. Perforation of atretic PVs using laser-assisted balloon valvuloplasty was developed by Qureshi et al. (16) in 1991, mechanical perforation using stiff guidewires was initially described by Latson et al. (10) in 1991, and radiofrequency perforation was initially described by Rosenthal et al. (17) in 1993. Subsequently, radiofrequency perforation has become the method of choice for transcatheter treatment of atretic PVs worldwide (18).

The current two-center study evaluated 62 neonates with PA/IVS and CPS who were treated over a 19-year period. Survival time until surgical intervention/valve replacement was analyzed as the endpoint with consideration of morphological and procedural data. In total, 15 patients (24.2%) reached the study endpoint by 2021. Although multiple studies have included catheterization as an endpoint, we believe that these procedures have been developed and established as an alternative strategy to avoid surgical intervention during the neonatal period. Therefore, surgery or valve replacement was defined as the endpoint, regardless of the number of catheterizations. Although the indications for surgical intervention and valve replacement differed slightly within the two centers, differences in long-term survival until surgical intervention and/or valve replacement according to procedural and morphological characteristics were still comparable.

The results showed significantly shorter times of freedom from surgery and valve replacement in the group with a one-leaflet morphology of the PV than in the groups with two- and three-leaflet morphology.

The mechanisms of the balloon valvuloplasty were initially assessed by Walls et al. (19) through direct inspection of the valve during surgery, revealing tearing of the valve raphes and leaflets and avulsion of the leaflets. Other studies have confirmed these findings by direct observation during surgical procedures and echocardiographic examinations of small sample groups (20–25). Valves can tear along the raphes, leading to separated cusps, and also through the cusps. Therefore, we assume that the raphes had a stabilizing effect on torn cusps, leading to lower valve regurgitation. The possible avulsion of a cusp did not appear to lead to regurgitation severe enough to require early valve replacement. Moderate PV regurgitation was well tolerated. Favorable valve morphology with respect to the number of cusps appears to be a positive predictor of long-term freedom from the need for surgical intervention. Current clinical practice involves setting a BAR from 1.2 to 1.4 to significantly reduce the pressure gradient and the probability of reintervention (26–28). The smaller the BAR, the smaller the risk of PV regurgitation, leading to further intervention. In our study, the length of time of freedom from surgical intervention or valve replacement was not significantly different between the BAR ≤1.2 and >1.2 groups. This may be attributed to the exclusive use of compliant balloons, such as the Tyshak II and VACS II, for balloon valvuloplasty. These balloons can only be inflated up to four bars according to the instructions for use and will maintain a waist if an oversized balloon is used. Thus, we assumed that the use of these balloons, even if oversized, avoided the complete destruction of the cusp anchoring.

The PV diameter and PV *Z*-scores were significantly smaller in the BAR >1.2 group. However, regurgitation was the primary indication for surgical intervention in the BAR >1.2 group, leading to five valve replacements and one monocusp implantation among the eight patients who needed surgical intervention. In contrast, stenosis was the major indication for surgical intervention in the BAR ≤1.2 group, leading to a need for transannular patch repair in four of the seven patients. A BAR of ≤1.2 leads to less regurgitation than does a BAR of >1.2 (27). While severe PV regurgitation is well tolerated in childhood, it has detrimental effects on RV function and exercise capacity and is associated with an increased risk of arrhythmia and sudden cardiac death (29). Thus, valve replacement is needed. In contrast, the current study and the postprocedural and long-term follow-ups reported by Pathak et al. (27) show no significant difference in the persistent gradient across the PV between those with a BAR of ≤1.2 and >1.2. The incidence of stenosis recurrence is higher in patients with a BAR of ≤1.2, and thus, a BAR of 1.2–1.25 is recommended to reduce the incidence of PV regurgitation. However, other studies have reported that a BAR of ≤1.2 was a risk factor for restenosis (28, 30, 31).

An immediate post-valvuloplasty PV gradient of >30 mmHg is a risk factor for reintervention (28, 31). In the current study, the time until surgical intervention/valve replacement was significantly shorter in patients with a persistent gradient across the PV of >40 mmHg. However, the time until surgical intervention/valve replacement was not significantly different between the patients with a persistent gradient of ≤20 mmHg and those with a persistent gradient of 21–40 mmHg. Notably, the patients with a gradient of ≤20 mmHg had a decrease in the time without surgical intervention at 15 years of age because they required valve replacement due to severe PV regurgitation. However, patients with a persisting gradient of >40 mmHg mostly underwent surgery for transannular patch repair due to stenosis in the first years of life. Consistent with the literature, our results showed that a small PV diameter was a risk factor for surgical interventions (28). A PV diameter *Z*-score of less than −1.5 was associated with a significantly reduced time of freedom until surgical intervention or valve replacement. In addition, the RVOT diameter and TV diameter differed between those with *Z*-scores of less than −1.5 and greater than or equal to −1.5. Thus, we assumed that the ventricles were smaller in patients with a small PV diameter (*Z*-score less than −1.5) than in those with a large PV diameter (Z-score greater than or equal to −1.5). However, the mean BAR was significantly higher in the patients with a smaller PV diameter. Notably, the hemodynamic effect of a smaller PV diameter and smaller RVs on the preprocedural left ventricular pressures was significantly less profound in the patients with a PV diameter *Z*-score of less than −1.5 than in those with a PV diameter Z-score of greater than or equal to −1.5.

This study had some limitations owing to its retrospective nature and small sample size. The small sample size restricted the evaluation of predictors of the outcomes of percutaneous balloon valvuloplasty in neonates with PA/IVS or CPS. Consequently, the calculated significances should rather be interpreted as associations. The study being conducted in two different centers led to slight differences in the indications for surgical intervention and valve replacement. Thus, long-term freedom from surgical intervention and/or valve replacement should be analyzed in larger samples in the future. Multicenter studies with more standardized procedures and indications for surgical interventions are also needed to improve the evaluation of the related parameters and procedural outcomes. In addition, RV function, which had a significant impact on outcomes, varied considerably and should be evaluated using longitudinal data.

In conclusion, percutaneous balloon valvuloplasty is a safe and effective treatment modality as an initial alternative for surgery such as shunt surgery or the Brock procedure in neonates with PA/IVS or CPS. Overall, the long-term outcomes are excellent, with favorable morphology and long freedom from the need for surgical intervention. A lower number of leaflets, small PV diameter, and a high persistent gradient >40 mmHg after balloon valvuloplasty all increase the risk of early surgical intervention. These findings provide a basis for accurately predicting the outcomes of balloon valvuloplasty in pulmonary atresia with intact ventricular septum and critical pulmonary valve stenosis. They establish the best timing for surgical intervention and provide the attending physician with a reference when counseling parents regarding outcomes and further procedures.

## Data Availability

The raw data supporting the conclusions of this article will be made available by the authors, without undue reservation.
